# Strategies for the Development of Industrial Fungal Producing Strains

**DOI:** 10.3390/jof9080834

**Published:** 2023-08-08

**Authors:** Sonia Salazar-Cerezo, Ronald P. de Vries, Sandra Garrigues

**Affiliations:** 1 Fungal Physiology, Westerdijk Fungal Biodiversity Institute & Fungal Molecular Physiology, Utrecht University, Uppsalalaan 8, 3584 CT Utrecht, The Netherlandsr.devries@wi.knaw.nl (R.P.d.V.); 2 Food Biotechnology Department, Instituto de Agroquímica y Tecnología de Alimentos (IATA), Consejo Superior de Investigaciones Científicas (CSIC), Catedrático Agustín Escardino Benlloch 7, 46980 Paterna, VLC, Spain

**Keywords:** fungal strain improvement, genetic engineering, fungal transformation, expression tools, recombinant DNA strategies, omics technologies, screening methods

## Abstract

The use of microorganisms in industry has enabled the (over)production of various compounds (e.g., primary and secondary metabolites, proteins and enzymes) that are relevant for the production of antibiotics, food, beverages, cosmetics, chemicals and biofuels, among others. Industrial strains are commonly obtained by conventional (non-GMO) strain improvement strategies and random screening and selection. However, recombinant DNA technology has made it possible to improve microbial strains by adding, deleting or modifying specific genes. Techniques such as genetic engineering and genome editing are contributing to the development of industrial production strains. Nevertheless, there is still significant room for further strain improvement. In this review, we will focus on classical and recent methods, tools and technologies used for the development of fungal production strains with the potential to be applied at an industrial scale. Additionally, the use of functional genomics, transcriptomics, proteomics and metabolomics together with the implementation of genetic manipulation techniques and expression tools will be discussed.

## 1. Introduction

**Figure 1:**
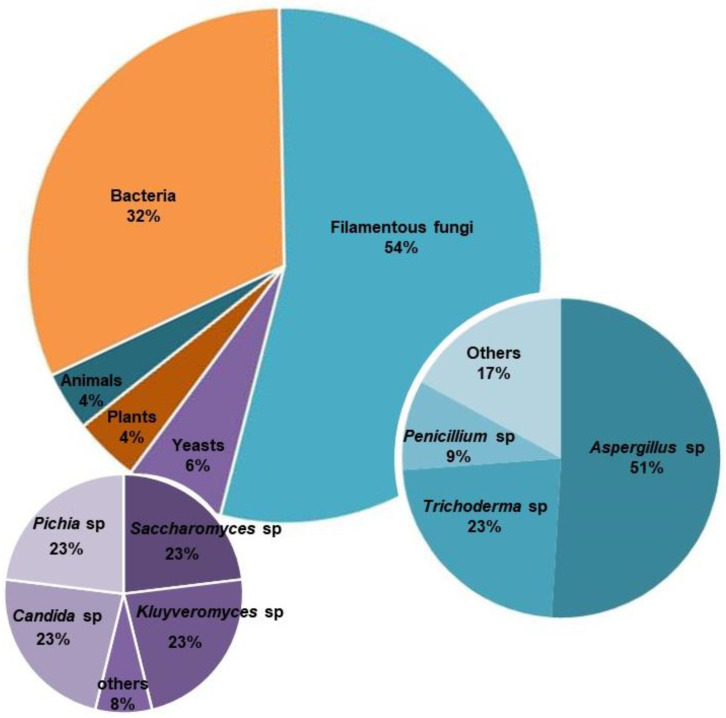
Main organism sources of commercial enzymes for biotechnological applications. Fungal origin is highlighted and detailed. Based on the list of commercial enzymes from the Association of Manufacturers and Formulators of Enzyme Products (AMFEP, 2015).

Historically, humanity has practiced rudimentary biotechnology without prior knowledge of the underlying biological mechanisms, e.g., for the production of beer, wine or bread. However, in the last decades, industrial biotechnology has changed drastically. Microorganisms are widely used in large-scale industrial processes as cell factories for the production of a variety of compounds, such as ethanol, organic acids, antibiotics, vitamins, proteins and enzymes [1], which have a wide variety of industrial applications (e.g., food, feed, biofuels, biochemicals, cosmetics, pharmaceuticals, textiles, pulp and paper and construction) [2]. Processes using bacteria, mammals and fungi have been widely used for production at an industrial level. In this review, we will focus on classical and recent methods used for the development of fungal producing strains. Whereas bacteria and yeasts have been accompanied by the development of sophisticated gene expression systems, filamentous fungi lag behind in providing comparable systems [3]. However, filamentous fungi are excellent candidate microbial cell factories. They naturally produce enzymes for the efficient decomposition and conversion of various kinds of biomass [4], especially plant biomass [5], and have a high protein secretion capacity, which is a common feature of their decomposing lifestyle. Fungal-based systems have several advantages over other (e.g., bacterial-based) systems. In addition to their high-level protein secretion capacity, they will be the vehicle of choice for large-scale production of recombinant proteins of eukaryotic origin, due to shared critical processes in gene expression and post-translational modifications with other eukaryotic organisms [6]. Fungi, particularly filamentous fungi, are used as expression hosts for proteins that require elaborate post-translational modification, e.g., protein glycosylation, proteolytic cleavage or multiple disulfide bond formation [7], which are key for the stability and activity of functional proteins. Yeast expression systems, such as *Pichia pastoris*, can secrete recombinant proteins with high efficiency and purity in many cases [8]. However, the secretory potential of filamentous fungi is reported to be higher than that of yeast strains [9]. In fact, fungal enzymes make up more than half of the enzymes currently used in industrial applications, with *Aspergillus, Trichoderma* and *Penicillium* being the most widely used filamentous fungal genera in industry [10] (Figure 1). Additionally, many filamentous fungal production strains have a GRAS (Generally Recognized As Safe) status [11], which is also a great advantage.

Although filamentous fungi are good candidate cell factories, they may also present certain drawbacks during protein synthesis. Filamentous fungi often modify their glycoproteins with heterogeneous high-mannose glycan structures, which can have undesired effects on the functionality of the resulting protein, especially for a therapeutic protein, which can negatively affect its pharmacokinetic behavior and reduce the efficiency of downstream processing. However, this problem can be solved by engineering the fungal glycosylation pathway to produce homogeneous and, even human-like, glycan structures [12]. Another drawback of some filamentous fungal strains is that the secretion of recombinant proteins is accompanied by the secretion of host proteins, which in many cases also include proteases, and this could (1) affect the subsequent purification steps and application of the target protein(s), and (2) result in degradation of the heterologous protein(s). The construction of low background host strains in filamentous fungi has been implemented [13,14]. However, the application of mutagenesis to obtain hosts with low background expression is labor intensive. In addition to this, naturally occurring low-protease-producing fungal strains, such as *Aspergillus vadensis,* have been reported as alternative fungal strains for heterologous protein production [15]. In any case, filamentous fungi have undoubtedly become major contributors to the production of the majority of microbial-derived industrial products [16] and have become essential contributors to the circular bio-economy [2]. In the food industry in particular, fungi have been traditionally used to produce fermented foods and beverages. Traditional fermentation processes include soy sauce or the production of alcohol from rice (yielding sake) by *Aspergillus oryzae*; blue cheese colonized by *Penicillium roqueforti*; and salami aged and seasoned via colonization by unique *Penicillium* species [17]. More recent efforts to utilize fungal-derived products have led to the development of new-generation products based, for example, on the production of single-cell proteins (SCP). Fungal SCP generally contain a protein content of 30–45% [18]. Among the SCP products, the ‘mycoprotein’—protein-rich food made of filamentous fungal biomass—can be consumed as an alternative to meat. Some of these SCP products include the Quorn (mycoprotein of *Fusarium venenatum*) and PEKILO (mycoprotein from *Paecilomyces variotii* [4,18]. Although filamentous fungi have many potential applications, improvement of the biosynthetic capabilities of industrially relevant fungal species to produce desired proteins, enzymes and metabolites in high quantities is one of the most important challenges of modern biotechnology. In this study, different strategies for the development of industrial fungal production strains are reviewed. Furthermore, advantages and limitations, as well as future prospects for strain development strategies are discussed.

## 2. Strain Improvement Strategies for Industrial Applications 

Strain development plays a key role in the industrial production of many compounds because it allows an organism to perform a biotechnological process more efficiently. Nowadays, there are multiple strategies to improve fungal characteristics (e.g., growth rate, substrate adaptation and utilization, stress resistance, etc.), which may result in higher production yields. Classical and genetic engineering are two commonly used approaches for strain improvement. However, while classical engineering does not require an in-depth understanding of the molecular basis of the manipulated microorganisms, genetic engineering allows a high level of control of the strain modifications. In this section, these two different strategies to improve fungal strains are described and their advantages as well as limitations are discussed.

### 2.1. Classical (Non-GMO) Strain Improvement Approaches

Classical strain improvement has long been regarded as the gold standard for fungal strain improvement in the industry because it can be applied even when there is limited knowledge about the genetic basis or biosynthetic pathways of the production organisms. Moreover, organisms obtained by classical mutagenesis are not subject to GMO legislation and can be used in the industry in the short-term [19]. Random (physical or chemical) mutagenesis and screening have been successfully performed in several filamentous fungi. As a result, many of the high-secreting mutants provide suitable strains for specific industrial goals [20], such as the overproduction of penicillin [21,22], and increased production of lignocellulolytic enzymes [23,24,25], lipases [26], citric acid [27] and bioethanol [28,29].

#### 2.1.1. Physical and Chemical Mutagenesis

Random mutagenesis is a rapid mutation-inducing technique that relies on the exposure of a microorganism to a physical or chemical mutagen in order to raise the frequency of mutation above the spontaneous rate [30]. The use of random mutagenesis has historically resulted in the production of strains with interesting characteristics for industrial applications. Physical mutagens, such as electromagnetic (e.g., rays, X rays and UV light) or particle radiation (fast and thermal neutrons, αand β particles), and chemical mutagens (N-Methyl-N′-nitro-N-nitrosoguanidine (NTG), ethyl methanesulfonate (EMS), 1-methyl-3-nitro-1-nitrosoguanidine (MNNG), sodium azide (NaN_3_) and nitrous acid (HNO_2_)) are often applied, alone or in combination, for fungal strain improvement [31]. More recently, Atmospheric and Room Temperature Plasma (ARTP) has been applied as a mutagenesis tool based on the radio frequency glow discharge of the atmospheric pressure [32]. In all cases, the type of mutations induced depends on two factors: the type of DNA damage caused by the mutagen and the action of the cellular DNA repair pathways on this damage. For example, far UV gives a high proportion of pyrimidine dimers. In contrast, ionizing radiation results in a high degree of chromosome breakage, whereas NTG and EMS are alkylating agents [33], and MNNG is a methylating agent [34]. In the case of ARTP, it can induce significant breakage of DNA strands with higher efficiency than chemical or UV mutagenesis at atmospheric pressure and room temperature (25–40 °C) [32]. However, its application in fungi is quite limited. Strain improvement by random mutagenesis is a successful method, but it is mainly a trial-and-error process. Changes are not directed exclusively at the loci that generate the beneficial change, which requires screening of large numbers of strains for the desired traits.

Random mutagenesis has been applied in a large number of fungal species for many industrial purposes, such as improved cellulase production in *Aspergillus* sp. [35], lipase production by *Aspergillus japonicus* [26] or citric acid overproduction by the industrial workhorse *Aspergillus niger* [36]. Moreover, UV-derived mutations were reported in *A. niger* to increase Filter Paper activity (FPase) and carboxymethyl cellulase (CMCase) production [37]. *Fusarium oxysporum* treated with UV followed by NTG also improved CMCase production [38], and exposure of *Pleurotus ostreatus* to UV caused an increase in laccase activity [39]. Random mutagenesis was also successfully applied on *Penicillium oxalicum* to enhance starch-degrading enzyme production [29] and for the generation of improved cellulase-producing strains. One of the cellulase-producing mutants, JU-A10-T, has been utilized for industrial-scale cellulase production since 1996 in China with a productivity of 160 U/L/h [40,41]. Other examples of industrial relevance are shown in Table 1.

#### 2.1.2. Adaptive Evolution

Another classical technique for strain improvement is adaptive evolution, also known as adaptive laboratory evolution, evolutionary engineering or whole-cell directed evolution. It relies on the basic principles of (natural and/or induced) genetic variation and subsequent strain selection. The technique involves the continued propagation of a microbial population under a desired selective pressure. Fitter mutants naturally arise from random mutations during DNA replication and increase in frequency in proportion to their fitness. Reliance on natural selection to enrich for mutants with increased fitness allows strain optimization to be performed without requiring prior knowledge of the genetic alteration(s) necessary to effect such changes. Adaptive evolution can also be combined with other methods such as random mutagenesis in order to generate more genetic diversity for selection [20,55]. Using adaptive evolution two thermotolerant variants of the entomopathogenic fungus *Metarhizium anisopliae* were obtained [56,57], which is one of the most successful and long-lasting biological control agents worldwide [58]. Adaptive evolution also allowed the generation of an *A*. *niger* strain with five-fold higher cellulase production than the original strain [59]. Growth on increasing levels of ferulic acid also resulted in an improved *A. niger* strain with a higher tolerance to aromatic compounds, which is beneficial for various industrial applications [60]. Adaptive evolution also improved inulinase production in *Aspergillus oryzae* [61]. In *Ashbya gossypii* ATCC 10895, adaptive evolution enhanced substrate (cane molasses) utilization and increased riboflavin yield. Results showed that riboflavin production increased by 97.5% and the dry cell weight increased by 125% compared with the parental strain [62].

Through this technique, an industrial *S*. *cerevisiae* strain, ISO12, with improved thermotolerance and tolerance towards hydrolysate-derived inhibitors was obtained. Contrary to the parental strain, ISO12 is able to grow and ferment non-detoxified lignocellulosic hydrolysates at higher temperature [63]. In the same species, strains obtained by adaptive evolution showed improved characteristics in laboratory-scale fermentations, such as enhanced fermentation rate, decreased formation of acetate and greater production of fermentative aroma [64]. In addition, adaptive evolution was used to optimize the brewer yeast towards a more flocculating phenotype. Flocculation or cell aggregation phenotype is a well-appreciated characteristic of industrial brewer strains since it allows the removal of the cells from beer in a cost-efficient and environmentally friendly manner. Furthermore, a transformant of *S. cerevisiae* carrying the genes from *Xanthophyllomyces dendrorhous* for β-carotene synthesis was subjected to adaptive laboratory evolution in combination with oxidative stress and selection of hyperproducing mutants, resulting in mutants with higher β-carotene synthesis [65].

#### 2.1.3. Protoplast Fusion

Protoplast fusion is another strategy for strain improvement that allows the genetic recombination and development of hybrid organisms. It requires the digestion of the fungal cell wall, which is frequently performed by specific enzymes. Exposure of the protoplast membrane allows for genetic manipulation that is less achievable with intact cells, such as cell fusion or the uptake of nucleic acids [66]. Protoplast fusion involves the generation of new strains by allowing genetic recombination between genomes of different parental strains, followed by selection of the strains showing the desired traits [67,68]. Protoplast fusion can be used to produce interspecific or even intergeneric hybrids [67]. Strains resulting from protoplast fusion of *T. reesei* and *A*. *niger* showed a three-fold increase of citric acid production in comparison with the parent *A. niger* strain [69]. Another intergeneric hybrid was obtained from *A*. *niger* and *Penicillium digitatum* for enhancing the production of verbenol, a highly valued food flavoring compound [70]. Fusant cells of *Aspergillus flavus* and *Aspergillus tamarii* produced higher amounts of ascorbic acid (8.85 g/L) compared to their parental strains (3.92 g/L and 4.57 g/L, respectively) [71]. Through protoplast fusion of two endophytic *Nodulisporium sylviforme* strains, several fusants were obtained. One of them produced 468.62 μg/L taxol, a diterpenoid with anticancer properties and production was increased up to 24.51% compared to the parental strains [72]. In *T. reesei*, fusants with higher CMCase activity were obtained, and more than two-fold increase in enzyme activity was observed compared to the parental strains [73]. Furthermore, in order to convert cellulosic materials to ethanol by a single step process, Kumari and Panda carried out protoplast fusion between *T*. *reesei* QM9414 and *S*. *cerevisie* NCIM 3288 [74]. The fusants produced ethanol directly from cellulosic materials [75]. Additionally, improvement has been reported for the fusion between the strain *S. cerevisiae* Q, frequently used as a beer producer, with *S. cerevisiae* L. The fusant showed higher ethanol tolerance (14% *v/v*) than the strain Q (10% *v/v*) [68]. The protoplast fusion of *S. cerevisiae* and *C*. *shehatae* followed by UV mutagenesis resulted in an increase of ethanol production at 42 °C, with 90% fermentation efficiency [76]. Finally, *Monascus ruber* and *Pleurotus ostreatus* were used as parent strains using the protoplast fusion technique. The new fusants were shown to significantly increase lovastatin content, a cholesterol-lowering drug [77].

#### 2.1.4. Genome Shuffling

The global demand for engineering complex phenotypes requires large-scale combinatorial approaches. The technology of genome shuffling has been shown as a genome engineering approach for the rapid improvement of strain phenotypes [78]. This approach uses recursive protoplast fusion with multi-parental strains to combine different mutations in the same cell, leading to additive or synergistic effects [79]. Although genome shuffling originates from protoplast fusion, they are considered different technologies. Traditional protoplast fusion is the fusion between two cells with different genetic traits. It leads to a stable recombinant strain with the combination of the genetic traits of both parents. In contrast, genome shuffling is the recombination between multiple parents of each generation, and several rounds of genome fusion are carried out. As a result, the final improved strains inherit the genetic traits from multiple initial strains [80]. Genome shuffling is time consuming, but its application does not require expensive facilities [78]. Furthermore, shuffled strains are not subject to GMO legislation and can, therefore, be directly used in the industry [80].

To improve lipase production in the phytopathogenic fungus *Penicillium expansum*, five mutants of *P. expansum* FS8486 were generated using NTG, which showed increased lipase production compared to the parental strain. These five mutants were subsequently combined with the wild-type strain of *A*. *tamarii* FS-132, and the lipase activity of one of the shuffled strains increased up to 317% compared to the starting strain FS8486 [81]. Genome shuffling had also been used to enhance cellulase production in *Trichoderma viride*. The strain obtained after two rounds of genome shuffling exhibited a total cellulase activity of 4.17 U/g, which was 1.97-fold higher than that of wild-type *T*. *viride* [82]. Additionally, a 100% improvement of cellulase production in *Penicillium decumbens* by genome shuffling has been reported [83]. Furthermore, it has been reported that after two rounds of genome shuffling in *Pichia anomala* TIB-x229, an improved variant designated as *P*. *anomala* GS2-3 was obtained. This strain could generate 23.1% higher D-arabitol yield than the original strain [84]. Genome shuffling has been also used to obtain better drug fungal producer strains. *Nodulisporium sylviform* NCEU-1 was used as starting strain to apply random mutagenesis and genome shuffling techniques in the breeding of taxol-producing fungi. After four cycles of genome shuffling, a mutant with high taxol production was obtained. The resulting strain showed 64.41% higher taxol production than that of the starting strain NCEU-1 [85].

Regarding the improvement of yeasts of industrial value, *S*. *cerevisiae* F34 strain was generated from the industrial yeast strain SM-3 after three rounds of genome shuffling, showing improved thermotolerance, ethanol tolerance and ethanol productivity [86]. Genome shuffling between *S*. *cerevisiae* and *Scheffersomyces stipites* (formerly *Pichia stipitis*) resulted in the yeast hybrid SP2-18, which showed more efficient substrate utilization compared to *S. cerevisiae* parental strain. SP2-18 was able to consume 34% of xylose present in the fermentation medium, whereas the *S. cerevisiae* strain was not able to efficiently utilize this sugar. Furthermore, SP2-18 was able to reach higher ethanol productivity (around 1.03 g/L) compared to the parental strain [87].

### 2.2. Genetic and Metabolic Engineering 

Since the advent of recombinant DNA technology in the 1980s, genetic engineering has been successfully implemented for the development of strains capable of (over)producing proteins, enzymes and other interesting metabolites [88]. In contrast to classical methods for strain improvement, genetic engineering allows a high level of control of the strain modification(s). Over the last decades, GMOs have revolutionized many fields, including medicine, agriculture, food and pharmaceutical industries [89]. However, due to social concern about the impact of GMOs on animal/human health and the environment, the application of genetic engineering for strain improvement is strictly controlled with an extensive legal framework, risk management and assessment procedures [90]. In the European Union, the two main legal instruments for GMO safety assessment are Council Directive 2001/18/EC, which provides the principles regulating the deliberate release of GMOs into the environment, and Regulation 1829/2003/EC, which strengthens and expands the rules for GMO safety assessment by introducing the ‘one-key-one-door’ approach and ensures the free movement of safe and healthy genetically modified products in the market [91]. In the US, GMO regulation is divided among three regulatory agencies: The Environmental Protection Agency (EPA), the Food and Drug Administration (FDA) and the US Department of Agriculture (USDA). The US approach to regulating GMOs is premised on the assumption that regulation should focus more on the nature of the final product, rather than the process in which it is produced, making GMO regulation in the US relatively favorable for their development. In other countries such as Australia, the import and use of GMOs are strictly regulated through a nationally consistent legal scheme, including the Commonwealth Gene Technology Act 2000, the Gene Technology Regulations 2001 and the corresponding state laws. South Africa has a fairly vigorous regulatory regime governing GMO use, including contained use, trial release, commercial release and transboundary movement. The primary legislation governing this issue is the Genetically Modified Organisms Act of 1997, together with a number of other laws imposing additional rules on GMO-related activities, including the National Environmental Management: Biodiversity Act, the Consumer Protection Act and the Foodstuffs, Cosmetics and Disinfectants Act. Clearly, different countries are seeing GMOs from distinct perspectives and are making rather different risk assessments as a result.

In the last decades, there has been much interest in exploiting the advances made in genome sequencing, comparative genomics and gene cloning for the design of metabolic pathways for the synthesis of specific compounds. Therefore, strategies beyond genetic engineering are often required. In this context, metabolic engineering provides an alternative and complementary method for strain improvement. Metabolic engineering is applied for the directed improvement of cellular properties through the modification of specific biochemical reactions or the introduction of genes [92]. Furthermore, the availability of genetic engineering tools such as expression vectors and transformation protocols are essential for metabolic engineering [88]. Metabolic engineering has been subject to continuous development. It is considered as a combination of multidisciplinary subjects built on principles from chemical engineering, computational sciences, biochemistry and molecular biology [93]. Recent advances in systems biology, the integration of experimental and computational research, and synthetic biology are allowing to apply metabolic engineering at the whole cell level, thus enabling the optimal design of microorganisms for the efficient production of drugs, cosmetic and food additives, among others [88,94]. 

## 3. Fungal Transformation Methods and Expression Tools for Strain Improvement

Genetic transformation represents a form of horizontal gene transfer and is defined as a process by which exogenous genetic material is taken up into a cell, which allows for increasing production levels, producing novel compounds or directing the synthesis of the desired products [95]. In fungi, this can only be achieved with the development of efficient transformation methods and expression tools. Introducing the desired genetic modifications in the fungus of interest often represents a challenge, since the establishment of an efficient transformation method might be difficult for many fungal species [96]. Nevertheless, all major groups of fungi can be transformed, and the genetic manipulation of these organisms is of great importance not only for research purposes but also for their applications in biotechnology [97]. To date, several transformation methods have been described for filamentous fungi, which include the commonly applied protoplast-mediated transformation, the *Agrobacterium tumefaciens*-mediated transformation and electroporation [98].

### 3.1. Protoplast Mediated Transformation (PMT)

The application of the protoplast-mediated transformation (PMT) has been extended to many filamentous fungi since the first successful transformation of the winemaking, baking and brewing yeast *S. cerevisiae* [99]. Protoplasts are cells from which the cell wall is removed, often by enzymatic digestion. Once protoplasts are generated, they are put into contact with the DNA of interest, and chemicals such as polyethylene glycol (PEG) are used to promote the fusion of the exogenous DNA and the protoplasts [96]. Finally, protoplasts are cultured on a selective medium. PMT has become one of the most commonly used methods for the transformation of filamentous fungi due to its simplicity and high efficiency [100,101]. It has been successfully applied in many filamentous fungal genera such as *Penicillium* [102,103,104,105,106,107], *Fusarium* [108] and *Aspergillus* [101,109,110,111], including the industrial workhorses *A. niger* [112,113,114] and *A. oryzae* [115,116]; and *Trichoderma*, including *T. reesei* [117,118], among others.

### 3.2. Agrobacterium tumefaciens Mediated Transformation (ATMT)

*Agrobacterium tumefaciens* is a Gram-negative soil bacterium that induces tumors in plants upon infection. It naturally carries a plasmid that contains the transfer DNA (T-DNA) flanked by two directional sequence repeats known as left and right borders [119]. This microbe has been largely studied as a biotechnological tool for the introduction of foreign genes into plants but can also be used for fungi. For transformation of filamentous fungi, a vector is designed in which the gene of interest is inserted between the left and right borders of the T-DNA, and *A. tumefaciens* is used as a vehicle to integrate the genes of interest into the fungal genome [96]. ATMT consists of three main steps: (i) *A. tumefaciens* induction for the expression of the necessary genes for the T-DNA transfer to the fungal cells, (ii) co-inoculation of the bacterium and fungal cells, and (iii) selection of the positive transformants with the appropriate selection pressure [120]. ATMT leads to single-copy integration, in contrast to PMT or electroporation (see Section 3.3), in which multi-copy integrations are often observed [96,101]. ATMT has been successfully applied in a range of filamentous fungi, such as the economically important *Aspergillus* sp. [101,121], *T. reesei* [121], phytopathogenic fungi such as *Botrytis cinerea* [122], *P. expansum* [123], *P. digitatum* [124], *F. oxysporum* [125] and *Pyricularia* (*Magnaporthe*) *oryzae* [126], *Neurospora crassa* [121] and in some basidiomycete and zygomycete species [120].

### 3.3. Electroporation

Electroporation is a fast and efficient transformation method that can be directly applied to both sporulating and non-sporulating fungal species [127]. It is based on the reversible capacity of cell membranes for permeabilization after the application of electric pulses. During permeabilization pores can be formed in the cell membrane, allowing the uptake of exogenous DNA [96]. After the electric pulse, once the DNA has entered the cells, biological membranes are restored, and DNA normally integrates into the genome. Compared to PMT and ATMT, electroporation is simpler and faster. Additionally, it allows the insertion of multiple copies of a given gene of interest, providing great potential for increased yields of the desired compounds. Nevertheless, instrumental costs hinder its applicability. Electroporation-mediated transformation has been applied in several fungal species such as *N. crassa, Penicillium urticae* [127], *Pseudogymnoascus verrucosus* [128], *Monascus purpureus* [129] or *T*. *harzianum* [130], and in some other filamentous fungi of industrial relevance such as *A. niger* [131], *A. oryzae* [127] or *T. reesei* [132].

### 3.4. Expression Tools

In the past decades, vectors have become pivotal expression tools in the field of molecular biology. Plasmids are, along with phages and integrative conjugative elements, the key vectors of horizontal gene transfer and essential tools in genetic engineering [133]. The developed vectors have been obtained through different technologies. The most commonly applied one has been the classic restriction enzyme and ligase-dependent cloning (RE&L). Furthermore, the development of techniques such as In-Fusion [134], USER [135], Gateway [136] and Golden Gate cloning [137,138] have simplified the vector experimental design.

Since the first design of a plasmid [139] and subsequent construction of the pBR322 vector [140] used as the base module for engineering, an enormous number of vectors has been reported. In yeast, the use of “shuttle vectors” are indispensable. They enable the cloning of defined DNA sequences in the bacterium *Escherichia coli* and their direct transfer into yeast cells. There are three types of commonly used yeast shuttle vectors: centromeric plasmids (YCps), episomal plasmids (YEps) and integrating plasmids (YIpS) [141]. The YCps need autonomously replicating sequences (ARS) and centromeric sequences (CEN) behaving like microchromosomes [142]. The YEps are based on sequences from a natural yeast plasmid. Meanwhile, YIps need to have homology sequences so they can integrate into the yeast genome [141]. Shuttle vectors have evolved over time. The first vectors were considerably larger and with few unique restriction sites for cloning. After that, smaller vectors with a higher number of MCS have been described e.g., the pRS series based on pBluescript, a vector with the MCS located within the *LacZ* gene [143], which allows blue-white screening (α-complementation). Furthermore, they presented different selection markers to choose from [144]. The pAG series contains more than 200 options of YCps, YEps and YIps vectors for cloning, with different expression and reporter genes such as green fluorescent protein (GFP) and red fluorescent protein (dsRed) [145]. Other examples include the collection of EasyClone, EasyClone2.0 and EasyCloneMulti vectors [146,147]. Recently, new vectors have been described to be used in new technologies, such as CRISPR/Cas9 genome editing [148,149]. 

In filamentous fungi, plasmids pALS-1, pALS-2 and pDV1001 were the first vectors shown to replicate autonomously in the nucleus or cytosol of a filamentous fungal species [150,151]. All were derived from *E. coli* plasmids. For example, the vector pALS-1 was based on the backbone of the mitochondrial plasmid P405-Labelle and on the *E. coli* plasmid pBR325 and contained the *Neurospora qa-2+* selection gene, which encodes a catabolic dehydroquinase (3-dehydroquinate hydro-lyase) [150]. Years later, Punt and coworkers reported the construction of the vectors pAN7-1 and pAN8-1 which confer resistance to the antibiotics hygromycin B and phleomycin, respectively [152,153]. Fernandez-Ábalos reported the adaptation of the GFP to be expressed in filamentous fungi as a reporter for gene expression [154]. Another vector described was pPgpd-DsRed. The pPgpd-DsRed vector was constructed by replacing the β-glucuronidase (*uidA*) gene in the fungal vector pNOM102 with the DsRed-Express gene sequence. This vector was used for co-transformation of *Penicillium paxilli*, *Trichoderma harzianum* and *Trichoderma virens* [155]. 

Furthermore, after Bundock et al. published the ATMT transformation as a novel method for the transformation of yeasts [156] and, subsequently, Groot et al. demonstrated that the ATMT system could be used for the transformation of several filamentous fungi [121], many vectors were constructed. To establish the ATMT transformation system, the pUR5750 vector was developed. Vectors for ATMT were derived from plant transformation vectors. The pCAMBIA vector series based on the pPZP series has been the most common starting material, followed by the original pPZP series, pBIN19, pGreen, pAg1, pCB301 and pBI121. The backbones of these binary vectors have typically not been subject to modification, whereas the T-DNA region is continuously being modified to be compatible with expression and functionality in the target fungal species. However, in a few cases, such as for pAg1, the backbone has been trimmed by removing nonessential structures from the pBIN19 backbone [157,158]. In addition, the development of CRISPR/Cas9-based vectors such as the pFC330 vector, with distinct fungal selectable markers (*pyrG*, *arbB*, *bleR* or *hygR*) [159] or Anep8_Cas9 plasmid [160] have been applied to fungal transformation. Nowadays, the number of vectors available for genetic transformation into filamentous fungi is enormous. For example, more than 180 binary vectors have been reported for ATMT [158]. In Table 2, some of the vectors used to transform fungal strains are enlisted, but the number of vectors available increases continuously.

## 4. Genetic Engineering-Based Methods for Rational Modification

Genetic engineering is a powerful approach that seeks the optimization of cellular processes. It allows increasing productivity and minimizing the formation of unwanted by-products. To achieve these goals, different methods for the rational modification of a given microorganism can be applied, from the classic molecular cloning and transformation approach to the recently developed CRISPR/Cas genome editing technology. 

### 4.1. Molecular Cloning

Molecular cloning methods are essential tools for biotechnology and also for biological research. Conventional methods usually require several restriction enzyme-mediated cloning steps to generate a construct of interest. However, alternative multipartite assembly methods have been developed based on the BioBrick/Phytobrick syntax, such as Golden Gate, the Golden Gate-based Modular Cloning (Mo Clo) or GoldenBraid/FungalBraid assemblies [181,182,183,184]. In these cases, genetic constructs can be assembled from standardized biological parts. Examples of DNA parts include promoters, signal peptides, ribosome-binding sites, coding sequences and transcriptional terminators. This is particularly useful when (re-)constructing metabolic pathways that are encoded by many genes and need to be assembled in various combinations to search for improved phenotypes [185]. 

Alternatives to these restriction/ligation-dependent methods include a number of overlapping extension techniques without the need for restriction enzymes. The Gibson Assembly^®^ method is a single-step cloning procedure that allows the cloning of two or more fragments through user-defined overlapping ends to allow a seamless joining [186]. Another strategy is called circular polymerase extension cloning (CPEC), which is based on polymerase overlap extension. The CPEC strategy is a simple method that has been successfully demonstrated with both multi-way parallel assembly and combinatorial library construction [187]. Other overlapping approaches include In-Fusion [188], Uracil-Specific Excision Reagent (USER) [189] and Sequence- and ligation-independent cloning (SLIC) [190]. These approaches are more applicable for plasmid or small pathway construction due to the drop in efficiency and rise in the error rate of PCR reactions as the product size gets larger [191].

### 4.2. CRISPR/Cas9 Technology

The CRISPR (clustered regularly interspaced short palindromic repeats)-Cas9 (CRISPR-associated nuclease 9) technology has dramatically changed the field of genome engineering since its first discovery in bacteria and archaea [192], receiving the Nobel Prize for Chemistry in 2020. The CRISPR/Cas9 system involves two main components: Cas9, which is a type II RNA-guided endonuclease, and the customizable and (re)programmable single guide RNA (gRNA) [193,194]. To induce site-specific genome editing, the gRNA targets the genomic DNA of interest via homology base pairing. A gRNA guides the endonuclease Cas9 to bind the target sequence to induce DNA double-strand breaks (DSBs) in the presence of the Protospacer Adjacent Motif (PAM), which needs to be located immediately downstream of the target sequence [195]. After cleavage, DSBs are recognized as potentially lethal damage that needs to be repaired to ensure the survival of the organism. In eukaryotic systems, including filamentous fungi, there are two ways to repair DNA damage: the error-prone non-homologous end-joining (NHEJ) and the high-fidelity homology-directed repair (HDR) pathways. In NHEJ, DSBs are directly ligated by the Ku70 and Ku80 heterodimeric protein complex, often leading to imprecise DNA repair and disruption of gene function [196]. In contrast, HDR occurs in the presence of a homologous DNA template (referred to as a repair template) via homologous recombination [197,198], which can be used to precisely introduce the DNA sequence of interest in the target organism.

Even though Cas9 from the bacterium *Streptococcus pyogenes* (spCas9) is the preferred Cas nuclease for genome editing due to the abundance of its target PAM throughout the genomes (5′-NGG-3′) [192], alternative Cas nucleases have been developed in order to increase the possibilities for genome editing. Examples of this are engineered Cas9 variants with higher specificity (e.g., eSp-Cas9, SpCas9-HF…), Cas9 homologs from other bacterial species with different PAM specificities, or alternative Cas proteins different from Cas9, such as Cas12a, C2c2, and MAD7 [199,200], with the last being a more convenient Cas protein at the industrial level due to the absence of intellectual property protection [200].

Application of the CRISPR/Cas technology in biotechnology creates innovative applications for the breeding of strains exhibiting the desired traits for specific industrial applications. In many cases, the introduction of one modification is not sufficient to improve a strain for industrial use, and thus editing of multiple traits is required to fine-tune metabolic networks of potential fungal cell factories. One possibility of introducing multiple genomic modifications with CRISPR/Cas9 is to perform iterative rounds of genome editing by recycling the system or by using alternative selection markers. The multiplexing capabilities of this system are currently being exploited with both Cas9 and more recently Cas12a [201,202]. In other cases, strain improvement goes beyond genetic modification, and non-editing applications using Cas9 have also emerged. Filamentous fungi are known to be a rich reservoir of interesting bioactive compounds, but most of the responsible biosynthetic gene clusters are transcriptionally silent under laboratory or industrial conditions. Therefore, another possibility to develop efficient fungal cell factories is to activate those gene clusters. In this context, a modified CRISPR/Cas9 system has been developed to activate the expression of silent gene clusters and its efficiency has been recently demonstrated in fungi [203], including the economically relevant penicillin producer fungus *Penicillium rubens* [204].

The first application of the CRISPR/Cas9 technology in filamentous fungi was in the industrially relevant *T. reesei* [205] and in six different *Aspergillus* species, including the industrial workhorse *A. niger* [159]. Nowadays, the CRISPR/Cas9 system enables the genetic improvement of a wide variety of filamentous fungal species, including *P. oryzae* [206], *N. crassa* [207], *A. oryzae* [208], *Aspergillus fumigatus* [110], *P. chrysogenum* [209], *Alternaria alternata* [210], *Beauveria bassiana* [211], *F. oxysporum* [212], *Fusarium solani* [213], *Fusarium fujikuroi* [214], *A. niger* [113], *Penicillium subrubescens* [106], *P. expansum, P. digitatum* [107] and the polykaryotic industrial fungus *Monascus purpureus* [215], among others. 

### 4.3. Other Strain Engineering Approaches

Although CRISPR/Cas9 is becoming one of the most applied genome-editing technologies for strain improvement, there are other genome editing strategies that expand the toolbox for the genetic engineering of filamentous fungi. Transcription activator-like effector (TALE) nucleases (TALENs) comprise a DNA-cleaving nuclease fused to a DNA-binding domain that can be easily engineered, so TALENs can target essentially any sequence. Like CRISPR/Cas9, this technique also enables site-specific DNA modifications [216]. TALEs are a group of special effector proteins, which contain N- and C-termini for localization and activation and a central domain for specific DNA binding. TALE binding to DNA is mediated by the central region that contains approx. 30 tandem repeats of around 35 amino acids [217]. The amino acid sequence of each tandem repeat does not vary, except for the two adjacent amino acids (known as repeat variable diresidues, RVD). Repeats with different RVDs recognize distinct DNA base pairs, allowing the development of this technology for directed mutagenesis when fusing customized TALE to endonucleases that cut the target DNA, triggering the DSBs DNA repair mechanisms [123]. This technology was first used in the filamentous fungus *P. oryzae* [206], and adapted to other fungi including *A. oryzae* [218] and *T. reesei* [219]. Recently, using TALENs as a disruption method together with exonuclease overexpression has allowed efficient gene editing in *Rhizopus oryzae* [220].

Zinc Fingers (ZFs) are small, compact DNA-binding proteins that have been successfully adopted as genome editing tools [221]. Naturally, each alpha helix of a ZF shows a unique amino acid sequence that interacts specifically with its corresponding DNA sequence. For strain engineering, ZFs are coupled with a restriction endonuclease (Zinc-finger nuclease, ZFN) to induce DSBs in the target sequence, and thus, induce the genetic modification of interest at remarkably high frequencies [222]. In *Aspergillus nidulans*, the use of ZFNs has helped to study the repression of carbon catabolites, mediated by CreA [223], and the regulation of the *amdS* gene, a gene that encodes an enzyme acetamidase, necessary for the catabolism of acetamide [224]. 

Although all these genome editing techniques allow the generation of specific genomic modifications more precisely than random mutagenesis, the European Court of Justice ruled in 2018 that organisms generated by directed mutagenesis techniques, e.g., CRISPR/Cas9, ZFNs or TALENs require the same treatment as any GMO in the European Union (Directive 2001/18/EC). In this context, meeting the obligations of the GMO Directive implies cost- and labor-intensive pre-market evaluations and long approval processes, which may decrease investments and limit commercialization.

## 5. Recombinant DNA Strategies

Genetic and metabolic engineering strategies have enabled improvements in yield and titer for a variety of valuable molecules produced naturally in fungi, as well as those produced heterologously. However, wild-type strains do not often produce these compounds at the levels required in industry, or do not produce the desired enzymes with the required properties or catalytic specificity. To overcome these problems, different genetic engineering approaches have been developed to improve the industrial potential of fungi.

### 5.1. Gene Downregulation or Inactivation

Gene downregulation or inactivation is defined as a genetic engineering approach in which one target gene is made inoperative in an organism. Gene downregulation can be achieved either via deletion, mutation or silencing of the target genes.

#### 5.1.1. Gene Deletion

Gene deletion or gene knockout is a powerful method to address genetic functionality. In order to completely delete a gene, double crossover homologous recombination events at the genomic level are required. In filamentous fungi, DNA integrates preferably via NHEJ, which results in low frequencies of homologous recombination [225]. To obtain gene deletion mutants with high homologous recombination efficiency, defective strains in NHEJ with improved site-specific recombination have been constructed by deletion of the Ku70 or Ku80-encoding genes in numerous filamentous fungi, including the model organisms *N. crassa* [226] and *A. nidulans* [227], the industrial workhorses *A. niger* [228], *A. oryzae* [229] and *T*. *reesei* [230], or the phytopathogenic fungi *P. oryzae* [231] and *P*. *digitatum* [232].

Gene deletions can be applied for the generation of strains with higher production of industrially relevant enzymes than the wild types for example by the inactivation of transcriptional repressor-encoding genes. The double deletion of the genes encoding the repressors CreA and CreB (*creA* and *creB*) in *A. oryzae* resulted in an increased production of α-amylases, xylanases and β-glucosidases [233], enzymes which are used in various industrial fields such as food, pharmaceuticals, textiles, detergents and pulp and paper [41,234]. A *creA* knockout strain of *Trichoderma orientalis* also enhanced enzyme production, specifically cellulases and hemicellulases [235]. In *T. reesei* deletion of *Cre2*, an orthologue of *creB*, resulted in increased cellulase activity [236]. In *T. reesei* deletion of *dmm2* (putative DNA methylation modulator-2) had a significant improvement in cellulose production activity (150–200%), when compared to the parental strain RUT-C30, in the presence of microcrystalline cellulose (Avicel) or lactose [237]. Additionally, the deletion of *ace1,* which encodes the negative transcriptional regulator ACE1 in the same species, resulted in an increased expression of all the main cellulase-encoding genes and two xylanase-encoding genes in sophorose- and cellulose-containing cultures [238]. Similarly, deletion of *bglR*, a beta-glucosidase regulator, contributed to improved cellulase production in *T. reesei, P*. *decumbens* and *P*. *oxalicum* [40,239,240]. 

Gene knockout strategies have also enabled improved production of interesting metabolites and inhibition of toxic metabolites in filamentous fungi by metabolic engineering. After deletion of two fumarate reductase and the mitochondrial fumarase genes (*Mtfr1* and *Mtfr2*) of *Myceliophthora thermophila*, the resulting strain exhibited a 2.33-fold increase in fumarate titer, which is widely used in the food and pharmaceutical industries [241]. In *A. fumigatus,* deletion of *veA* and *laeA*, both encoding velvet complex components, up-regulated the gene cluster responsible for the synthesis of fumagillin [242]. Fumagillin has been intensely studied due to its potential in the treatment of amebiasis, microsporidiosis and for its anti-angiogenic activity as inhibitor of the human type 2 methionine aminopeptidase [242]. Deletion of the L-galactonic acid dehydratase-encoding genes *gaaB* and *lgd1* in *A. niger* and *T. reesei*, respectively, increased extracellular accumulation of L-galactonic acid, with potential applications in the pharmaceutical, cosmetic and other industries [243]. Deletion of the fifteen genes involved in the patulin biosynthetic pathway resulted in a decreased ability of *P. expansum* to produce patulin, a mycotoxin that can be present as a contaminant in food, particularly in fruits and fruit-derived products [244]. Meanwhile, in *S. cerevisiae* simultaneous deletion of *GPD2* (glycerol 3-phosphate dehydrogenase 2), *FPS1* (Aquaglyceroporin FPS1) and *ADH2* (alcohol dehydrogenase 2) increase ethanol production by 0.18% in comparison with the wild-type strain [245].

#### 5.1.2. Point Mutations

Gene inactivation can also be achieved by single DNA base pair deletion and/or single nucleotide changes. Point mutations can result in the exchange of nucleotides (substitution), elimination of nucleotides (deletions) or introduction of nucleotides in the DNA sequence (insertions). Point mutations are the most common source of genetic variation, and although most are neutral or deleterious, some become beneficial for the organisms, giving them novel characteristics, e.g., better adaptation to the environment or improvement of their catalytic performance. Point mutations in filamentous fungi are usually obtained using physical or chemical mutagens [246] (see Section 2.1.1). However, they can also be generated through side-directed mutagenesis, which allows the precise introduction of the target point mutations. 

As examples of industrial relevance, a point mutation in the hemi-cellulolytic transcriptional activator Xyr1 introduced via UV mutagenesis in *T. reesei* was found to result in a constitutively active form of this regulator, resulting in constitutive expression of cellulase and xylanase encoding genes, even in the presence of a repressing carbon source [247]. In addition, many *T. reesei* strains that are used in industry underwent point mutations leading to catabolite de-repression, resulting in increased extracellular enzyme and protein levels compared to their parent strain [24]. Point mutations also resulted in improved cysteine biosynthesis in *P. rubens* by the inactivation of enzymatic conversions that compete with the cysteine biosynthetic pathway, which plays a key role in penicillin production [248]. By site-directed mutagenesis, the thermostability of an *A. niger* xylanase has been improved, showing up to 80% of its maximal activity after incubation for 2 h at 50 °C in the presence of xylan, compared to only 15% activity for the wild-type enzyme [249]. Additionally, using the CRISPR/Cas9 system, a modified *gaaR* gene carrying a single point mutation causing a W361R amino acid change was introduced in *A. niger*, which causes constitutive activation of GaaR and therefore constitutive production of pectinases under non-inducing conditions [113,250].

#### 5.1.3. RNA Interference (RNAi)

The possibility to inactivate genes or metabolic pathways is not restricted to DNA-based approaches. RNA interference (RNAi) is an evolutionarily conserved mechanism found in most eukaryotic organisms, including fungi. It was originally described as a mechanism that confers protection against exogenous and endogenous genetic threats (virus, transposons...) and regulates gene expression by means of non-coding RNA of around 30 nucleotides, mainly short interfering RNAs (siRNAs), microRNAs (miRNAs) and piwi-interacting RNAs (piRNAs) [251,252]. This mechanism has been adapted as a potential biotechnological tool to improve fungal strains. It is particularly advantageous when target genes are present in multiple copies or when deletion of the target gene(s) is lethal. The resulting knock-down transformants still carry the target gene; however, RNAi leads to a reduction of the transcription level, which can be close to zero in some transformants [253]. Double-stranded RNA (dsRNA) induces the inactivation of cognate sequences by mRNA degradation, translation inhibition, chromatin remodeling or DNA elimination [254]. 

RNAi strategy was used to attenuate the expression of the *creA* gene in *P. chrysogenum* for higher penicillin production [255]. In *A. oryzae*, RNAi-based inactivation of three α-amylase encoding genes improved heterologous protein production in this species [256]. In *A*. *niger*, RNAi was used to knockdown chitin synthase activator (CHS3). *A. niger chs3* mutants exhibited better citric acid production potential compared to that of the parent strain in scale-up fermentation [257]. RNAi was also applied to silence the expression of hydroxymethyl glutaryl coenzyme A reductase (*hmgR*) and farnesyl pyrophosphate synthase (*fpps*) genes in *Fusarium* sp., resulting in higher levels of bikaverin, a known antimicrobial and antitumor compound [258]. 

### 5.2. Gene Up-Regulation 

Gene up-regulation is a genetic engineering approach aimed to increase the expression level of a target gene. In filamentous fungi, up-regulation of specific genes may increase the production of a metabolite or enzyme of interest or improve the conversion rate of a given substrate. Additionally, greater production of metabolites or enzymes can be achieved by the overexpression of the genes encoding regulatory proteins that control the expression of the corresponding genes. In the next sections, different gene up-regulation strategies in filamentous fungi for different purposes are reviewed.

#### 5.2.1. Promoter Swap

Gene expression in eukaryotic organisms is mainly governed at the level of transcriptional initiation, which corresponds to the complex interplay between the promoter, RNA polymerase II and transcription factors [259]. There are different types of promoter sequences. Constitutive promoters are always active regardless of environmental or internal signals, leading to overexpression of their target genes, and thus resulting in high production titers. In contrast, inducible promoters are controlled by transcription factors after the recognition of specific environmental signals, e.g., pH, presence of sugars or catabolic enzymes [260]. One possible genetic approach to (over)produce enzymes or specific metabolites relies on the substitution of the original promoter sequences for a constitutively active one. However, overexpression of some genes might lead to overburdening of the cellular mechanisms or the accumulation of (toxic) side compounds, which can be detrimental to cells. In this context, inducible promoters are a great alternative to control gene expression over time [260] and are the preferred choices at the industrial level, where a fine-tunable expression with cost-efficient induction is desired [7].

Promoter sequences of industrial relevance are for example the constitutively active promoter from the glycer-aldehyde-3-phosphate dehydrogenase (*gpdA*) from *A. nidulans* [261], or the inducible cellobiohydrolase I (*cbh1*) gene promoter from *T. reesei*. P*cbh1* is strongly induced in the presence of cellulose and has been widely applied for heterologous protein production in *T. reesei* and other filamentous fungi [262]. The promoter of the glucoamylase A gene (*glaA*) from *A. niger* was one of the first inducible systems applied in filamentous fungi, and drives gene expression in the presence of starch and starch-related compounds, while xylose represses gene expression [263]. P*glaA* has been reported to work efficiently in *A. terreus* to overexpress acetyl-CoA carboxylase for the increase of lovastatin production, a cholesterol-lowering compound [264]. In *A. oryzae*, the *glaA* promoter was used for the production of L-malate, a flavor enhancer which is widely utilized in the food and beverage industries [219]. Xylose-inducible promoters have also been established in some industrial hosts, such as the *xyn1* promoter from *T. reesei* and *xylP* from *P. chrysogenum* [265]. Another inducible promoter is the thiamine-regulatable *thiA* promoter, which was established in *A. oryzae* and is controlled by different concentrations of thiamine in the medium culture [266]. In the industrial workhorse *A. niger,* a promoter system was established in order to adapt to the medium conditions during citric acid fermentation [267]. During the process, the pH decreases dramatically, and therefore, the pH-responsive promoter from the 1,3-β-glucanosyltransferase GelD (P*gas*) was used, which enhanced gene expression at very low pH. P*gas* was also used to express the cis-aconitate decarboxylase encoding gene from *Aspergillus terreus* in *A. niger*. Since *A. niger* produces large amounts of citrate, which is the precursor of itaconate, the aim was to modify the natural citrate producer into an itaconate producer, with high potential for the production of resins, plastics, paints and fibers. Furthermore, itaconate, which is an immunomodulatory metabolite highly expressed in activated macrophages, has potential application in the treatment of inflammatory diseases [268]. The use of the P*gas* promoter led to a gradually increased production of itaconate, correlating with decreasing pH values [267]. The *xylP* promoter (P*xylP*) controlling expression of a xylanase from *P. chrysogenum* allows high induction by xylose and xylan with low basal activity in the absence of the inducer. P*xylP* was demonstrated to permit conditional gene expression of diverse genes in various mold species including *P. chrysogenum*, *A*. *nidulans* and *Aspergillus fumigatus*, among others. In *A. fumigatus*, it has been shown that P*xylP* mediates not only inducer-dependent activation but also repression in the absence of inducer. Furthermore, P*xylP* was found to act bi-bidirectionally with a similar regulatory pattern by driving expression of the upstream-located arabinofuranosidase gene. The latter opens the possibility of dual bidirectional use of P*xylP* [269].

An alternative type of promoters are bidirectional promoters (BDPs), which allow the expression of two genes at the same time, such as the gene of interest and the selection marker, two copies of the gene of interest, or two different genes. Recently, natural BDPs have been found in filamentous fungi. It has been shown that BDPs are always intergenic regions regulating the flanking two genes that encode proteins relevant to the same biological process [270]. The histone H4.1 and H3 promoters can act as natural BDPs, allowing simultaneous expression of enzyme encoding genes in the case of metabolic engineering of *Aspergillus* sp. [271]. Additionally, *A. niger* D-galacturonic acid reductase promoter shows bidirectional transcription, thus allowing its application as both bidirectional and inducible promoter [272]. In *T*. *reesei* a 767-bp intergenic region served as a bidirectional promoter. This region was shown to be able to drive the simultaneous expression of two fluorescence reporter genes when fused to each end. This promoter enabled *T. reesei* to produce cellulases on glucose and improved the total cellulase activities with cellulose Avicel as the sole carbon source [270]. 

In some cases, yeast expression systems have been shown to work more efficiently for the biotechnical production of mammalian proteins with pharmaceutical relevance. Common hosts are *S. cerevisiae*, *P. pastoris* and *Hansenula polymorpha* [273,274,275]. However, filamentous fungi are still used for the heterologous production of different mammalian proteins, for instance, the human hormone peptide obestatin using recombinant *T. reesei* strains [276].

#### 5.2.2. Increase of Gene Copy Number

Increasing gene copy number often results in increased protein production [277]. High gene copy numbers can be achieved by either adding high amounts of DNA during transformation; applying a strong antibiotic pressure during selection; or using bidirectional promoters. However, genomic loci can affect gene expression, leading to some extra copies remaining silent [278]. Moreover, increasing the number of copies can sometimes compromise the stability of the resulting strains, which is especially undesired in industries where prolonged cultivations are needed [279]. This strategy has been applied for instance in the production of penicillin by inserting multiple copies of the penicillin biosynthesis cluster in the genome of *P. chrysogenum* [280]. Similarly, increasing copies of the *glaA* gene in *A. niger* from one to twenty resulted in an increase of secreted glucoamylase levels [281]. Alternative ways of inducing high protein production can be associated not only with increased gene copy number but also with increased promoter strength through a multi-copy strategy. Enhancement of promoter strength was achieved when five copies of the −427 to −331 upstream region of *glaA* gene from *A. niger* were integrated to efficiently increase L-malate production directly from corn starch [282].

In order to achieve heterologous β-carotene synthesis in *Y*. *lipolytica*, which cannot indigenously produce β-carotene, the structural genes responsible for β-carotene synthesis were overexpressed. β-carotene is a kind of high-value tetraterpene compound, which shows various applications in medical, agricultural and industrial areas owing to its antioxidant, antitumor and anti-inflammatory activities. The strain obtained (*Y*. *lipolytica*-C (Yli-C)) reached 34.5 mg/L β-carotene [283].

## 6. Incorporation of Omics Technologies into Fungal Strain Improvement

Rapid improvements in sequencing technologies have greatly increased the number of fully sequenced fungal genomes available. In addition to the genome sequence data, related ‘omics’ approaches (transcriptomics, proteomics and metabolomics) coupled with bioinformatic analyses allow access to a striking variety of potential genes to target for downstream characterization and incorporation into bioproduction strategies for strain improvement [284]. 

Analysis of the genomes of industrial filamentous fungi has unraveled previously unknown enzymes, especially those involved in carbohydrate metabolism (CAZy) [285], but also proteases, lipases, and others. For example, in *T. reesei,* re-annotation of the CAZy encoding genes together with gene expression analysis in different culture conditions has evidenced the importance of several enzymes until then uncharacterized and provided additional information on the enzyme sets needed for the complete degradation of different lignocellulose substrates [286]. An in-depth analysis of the *T*. *asperellum* ND-1 genome suggested a unique enzymatic system, especially hemicellulases and chitinases. After a comparative analysis of lignocellulase activities of ND-1 and other fungi, it was found that ND-1 displayed higher hemicellulases (particularly xylanases) and comparable cellulases activities [287]. Moreover, it has revealed high variation in fungal enzyme sets produced by *Aspergillus* species during growth on complex plant biomass, despite the similarity among their genome sequences [288]. The analysis of genomic data from *A. niger* has also unraveled many genes and gene clusters involved in secondary metabolite production, illustrating its potential as a cell factory [289]. The development of genome sequencing technologies, especially with the establishment of genome mining, has enabled us to obtain new natural drugs in a faster and cheaper manner [290]. 

In addition, the use of transcriptomics together with other technologies allows the development of efficient metabolite-producing strains. Transcriptomic profiling of *Podoscypha petalodes* strain GGF6, a basidiomycete fungus that produces endocellulase, laccase and other lignocellulolytic enzymes under submerged fermentation conditions reveals the presence of 280 CAZy proteins. Furthermore, bioprospecting transcriptome signatures in the fungus revealed a diverse array of proteins associated with cellulose, hemicellulose, pectin and lignin degradation, including two copper-dependent lytic polysaccharide monooxygenases (AA14) and one pyrroloquinolinequinone-dependent oxidoreductase (AA12) which are known to help in the lignocellulosic plant biomass degradation [291]. In *A*. *niger,* a transcriptomics approach was used to identify genes involved in galactaric acid catabolism, and they were deleted using CRISPR/Cas9. As a result, an engineered *A. niger* strain was able to produce galactaric acid by the endogenous inactivation of the pathway for D-galacturonic acid catabolism [292]. Galactaric acid is used in skincare products or can be chemically converted for polymer production with different industrial applications [293]. Comparative transcriptomic analysis of a taxol-producing *Aspergillus aculeatinus* Tax-6 and its mutant BT-2 (an improved taxol-producing strain) showed up-regulation of the genes related to the mevalonate pathway, including geranylgeranyl diphosphate synthase-encoding gene [294].

Among the ‘omics’ techniques, proteomics is a powerful tool to identify hundreds of intracellular and extracellular proteins and, therefore, helps in the understanding of the molecular events that occur within an organism [295]. For example, the comparative proteomics analysis between two efficient cellulolytic strains of *T. reesei* (CL847 and RUT-C30) grown in media containing lactose or lactose-xylose revealed their different protein profiles. The proteins identified from the strain CL847 after growth in lactose-xylose medium included the major cellulases secreted by *T. reesei* (Cel7A and Cel6A), as well as β-xylosidases, xylanases and arabinofuranosidases. The strain RUT-C30 was unable to produce cellulases in the same medium. However, in the presence of lactose, the RUT-C30 secretome contained 10% higher level of cellobiohydrolases than the CL847 secretome [296]. A similar analysis was carried out between the fungi *T*. *reesei* and *A*. *niger,* and results also revealed differences in the enzyme set production [297]. As another example, a comparative transcriptomics and proteomics approach to identify differential gene expression and protein production of two commercial wine yeast (*S*. *cerevisiae* ICV 16 and ICV 27) during alcoholic fermentation was performed [298]. Results showed differences related to carbohydrate metabolism, nitrogen catabolite repression, and response to stimuli, among other factors. In addition, a relative increase in the abundance of proteins involved in stress responses (e.g., heat shock proteins) and in the fermentation process (e.g., the major cytosolic aldehyde dehydrogenase Ald6p) was observed in the strain with better behavior during vinification. Using metabolic engineering and a systems biology approach, a *S*. *cerevisiae* strain able to produce 242 mg/L of p-coumaric acid from xylose was reported [299]. The same strain produced only 5.35 mg/L when cultivated with glucose as sole carbon source. To characterize this strain further, transcriptomic analysis was performed, comparing the strain’s growth on xylose and glucose [299]. Integrative analysis of the transcriptome and proteome of *Trichoderma longibrachiatum* LC and two cellulase hyper-producing mutants were carried out to identify the candidate genes that regulated the cellulolytic enzyme synthesis and secretion processes. The integrative analysis of transcriptome and proteome showed that the protein processing in ER involved in the protein secretory pathway, starch and sucrose metabolism pathway and N-glycan biosynthesis pathway were significantly changed in the cellulase hyper-producing mutants, which may be the main reason for cellulase hyper-production in the mutants [84]. This information is important for a better understanding of the physiological differences between strains during industrial processes and for the identification of features that could contribute to the adequate adaptation of these strains. 

## 7. Strain Screening Methods 

To identify a successfully developed strain, efficient ways for mutant screening are needed. Screening strategies are divided into two basic types: non-selective random screening, in which randomly picked isolates are tested for the desired qualities; and rational selection, a method based on prior knowledge of the metabolism and regulation pathways of a microorganism, so the identification is carried out in a targeted manner [246,300]. In random screening, after inducing mutation survivors are randomly picked and tested for their traits of interest or their ability to produce the metabolite of interest. Screening a large number of mutated organisms usually identifies improved mutants. However, mutants with very high yields are rarer than those with small improvements, which makes this procedure repetitive, time consuming and labor intensive. As an alternative, the use of rational selection allows the identification of a higher number of desirable mutants in less time [246]. In rational screening, some basic understanding of product metabolism and pathways regulation is required. For example, some environmental conditions (pH, temperature and aeration) or chemicals can be incorporated into the medium. The use of analogue molecules to the metabolites of interest has allowed the identification of producer strains, for example, the fungi, *P. chrysogenum* and *Acremonium chrysogenum* are producers of the β-lactamic antibiotics penicillin and cephalosporin, respectively, which are derived from amino acid precursors. Mutants resistant to analogs of lysine and methionine resulted in higher production yields [301]. In *P*. *chrysogenum,* the addition of phenylacetic acid, a toxic precursor of penicillin, to the medium allows the selection of mutants with higher penicillin production [302]. In *A. nidulans*, the use of chlorate allows the selection of strains affected in nitrate reductase activity, encoded by the *niaD* gene. The isolated mutants are unable to use nitrate as a sole nitrogen source [303]. Finally, the use of congo red in the detection of fungal cellulolytic activity was reported in *Aspergillus* species [304]. The use of congo red as an indicator for β-D glucan degradation in an agar medium provides the basis for a rapid and sensitive screening test for cellulolytic microorganisms [305]. 

An alternative to the rational screening method is the use of marker genes that complement specific nutritional requirements (auxotrophy). Some of the most commonly applied marker genes are wild-type alleles of genes that encode key enzymes in the metabolic pathways toward essential monomers used in biosynthesis [306]. An example is the orotidine-5′-phosphate decarboxylase-encoding gene *pyrG* (a homologue of the *S*. *cerevisiae ura3* gene). Mutants that lack *pyrG* are auxotrophic for uracil, so vectors containing *pyrG* allow selection on uracil-deficient media. Additionally, *pyrG*-deficient mutants are resistant to 5-fluoro-orotic acid, which is toxic in prototrophs, allowing negative selection. Another gene used as a selection marker is the *amdS* gene. It encodes an acetamidase enzyme that hydrolyses acetamide. This allows acetamide to be used as a sole nitrogen or carbon source. It has been used as a dominant selection marker in many species of filamentous fungi like *Aspergillus awamori* [307], *A. niger* [308], and *T. reesei* [117] as well as in some yeasts, such as *Kluyveromyces lactis*, *S*. *cerevisiae* and *P. pastoris* [309]. The use of these genes as selection markers is restricted to host strains that are auxotrophic for the nutrient in question due to the absence of a functional chromosomal copy of the marker gene. Unless transformed to prototrophy with a functional allele of the marker gene, auxotrophic yeast strains can be propagated only in media that contain the appropriate growth factor(s) [306].

Finally, genes conferring resistance to antibiotics have been widely used as markers for the selection of transformed cells. The most widely used marker system is the antibiotic resistance to hygromycin [310,311], but also other antibiotics markers are used, such as bialaphos/phosphinothricin, geneticin/neomicyn, phleomycin and kanamycin resistance [312,313,314].

## 8. Conclusions and Future Perspectives

In industry, filamentous fungi have acquired a prominent position as producers of economically relevant proteins, enzymes, and primary or secondary metabolites due to their high capacity to perform as cell factories. In the food industry in particular, filamentous fungi have become important players in the production of new-generation foods. Fungal-based systems have several advantages over bacterial- and yeast-based systems to become cell factories. However, since many of them are usually not efficient enough to result in economically sustainable industrial processes, strain improvement strategies are needed to increase their productivity, reduce by-products and increase the tolerance to process conditions. Non-GMO approaches have the widest applicability at the industrial level since they are not subject to GMO regulations that limit the use of GMO-based approaches. However, these are unspecific, laborious and time-consuming. Genetic engineering of filamentous fungi has become an established approach in biotechnology, overcoming many of the disadvantages attributed to the classical strain improvement methods. Furthermore, the development of new genome editing technologies, such as CRISPR/Cas9, together with the implementation of different omics technologies (genomics, transcriptomics, proteomics, and metabolomics) has boosted the development of industrial strains with improved production capacities and performance. Increasing product demand and changes in consumer preferences are leading to increased interest in improving fungal cell factories. A better understanding of fungal metabolism together with the development of new strain improvement technologies and optimization of the currently available ones will help us face the increasing demand in the biotechnological industry.

## Figures and Tables

**Table 1:** Examples of improved strains by random mutagenesis.

Organism	Improvement (% Increase)	Method *	References
*Aspergillus brasiliensis*	Xylanase activity (36.3%)	UV mutagenesis Chemical mutagenesis (NTG and EMS)	[42]
*Aspergillus japonicus*	Lipase activity (177%)	UV mutagenesis Chemical mutagenesis (HNO_2_ or NTG)	[26]
*Aspergillus niger*	Cellulase production and carboxymethyl cellulase (CMCase) activity (100%)	UV mutagenesis	[37]
*A. niger*	Citric acid overproduction (60.25%)	UV mutagenesis	[36]
*Aspergillus oryzae*	Phytase activity (95%)	Chemical mutagenesis (sodium azide, nitrous acid and EMS)	[43]
*Aspergillus* sp.	Amylase activity (60.85%)	UV mutagenesis Chemical mutagenesis (HNO_2_)	[44]
*Aspergillus* sp.	Cellulase production (CMCase activity) (160%)	γ irradiation (Co^60^) UV mutagenesis Chemical mutagenesis (NTG)	[35]
*Aspergillus uvarum*	Cellulase production (CMCase activity) (90%)	Chemical mutagenesis (EMS)	[45]
*Candida shehatae*	Ethanol production (13%) and tolerance	UV mutagenesis	[46]
*Fusarium oxysporum*	CMCase activity (150%)	UV mutagenesis Chemical mutagenesis (NTG)	[38]
*Geotrichum candidum*	Pectinase production (100%)	UV mutagenesis Chemical mutagenesis (EtBr)	[47]
*Kluyveromyces marxianus*	Ethanol production (13%) and tolerance	UV mutagenesis	[48]
*Penicillium chrysogenum*	Penicillin production (100%)	X-ray mutagenesis, UV mutagenesis, Nitrogen mustard mutagenesis	[22,49]
*Penicillium digitatum*	Xylanase activity (15%)	UV mutagenesis, Chemical mutagenesis (NTG and EMS)	[42]
*Penicillium oxalicum*	Raw starch-degrading enzyme (RSDE) production (42.4%)	γ irradiation (Co^60^) Chemical mutagenesis (EMS) Genetic engineering (TF-based)	[29]
*Penicillium oxalicum* JU-A10-T	Cellulase production (36%)	UV mutagenesis, Chemical mutagenesis (NTG)	[40]
*Pichia stipitis*	Ethanol production (70%) and tolerance	UV mutagenesis	[50]
*Pleurotus ostreatus*	Laccase activity (77%)	UV mutagenesis	[39]
*Saccharomyces cerevisiae*	Ethanol production (13.2–25%) and tolerance	UV mutagenesis	[51,52]
*S. cerevisiae*	Ethanol production (81.02%) and tolerance	Atmospheric and room temperature plasma (ARTP)	[6]
*S. cerevisiae*	Amylase activity (250%)	UV mutagenesis	[53]
*Talaromyces pinophilus*	Cellulase production (28%)	UV mutagenesis Chemical mutagenesis (NTG and EMS)	[54]
*Trichoderma reesei*	Cellulase production (250%)	UV mutagenesis Chemical mutagenesis (NTG)	[9]

* The methodology and the order in which they were used varies in each case and in many of them the strains were subjected to several mutation steps.

**Table 2:** Examples of transformation vectors suitable for fungal genetic modification.

Plasmid Name	Fungal Selection *	Vector Size	Features/Description	Host	References
pBluescript II SK/KS (+)		3 kb	Standard cloning vector. pBluescript II SK(+) and pBluescript II KS(+) differ by the orientation of the MCS. Used as base for pRS fungal vectors	Bacteria	[143]
YXplac series	LEU2, URA3, TRPI	~4 to 5 kb	A series of nine vectors derived from pUC19, including YCps, YEps and YipS vectors	Yeast	[161]
pRS	HIS3, TRPI, LEU2, URA3	≤6 kb	A set of YCp and YIp vectors (pRS series) based on the pBLUESCRIPT	Yeast	[162]
pRS420	HIS3, TRP1, LEU2, URA3	6 kb	Plasmids based on the pRS plasmid, YEp-type vectors	Yeast	[163]
pRSII	ADE2, HIS2, HIS3, TRP1, LEU2, URA3, ADE1	5 kb	42 plasmids based on the pRS series, integrative plasmid	Yeast	[144]
pAG	HIS3, LEU2, TRP1, URA3	7 kb	A series of more than 200 options; contains fluorescence reporters. Gateway compatible	Yeast	[145]
pXP	URA3, TRP1, MET15, LEU2, HIS3, CAN1	5 kb	A series of 28 vectors, using expression of luciferase reporters	Yeast	[164]
EasyClone	HIS3, LEU2, LYS5, URA3	6 kb	Multiple integrations; recycling of markers	Yeast	[146]
EasyClone2.0	*amds, ble, dsd, hph, kan, nat*	6 kb	Recycling markers, integrative vectors suitable for (over)expression	Yeast	[165]
EasyCloneMulti	Kl.URA3-degradation signal	6 kb	Integrates into Ty sequences; recycling of markers.	Yeast	[147]
pRG	HIS3, LEU2, LYS2, MET15, URA3	6 kb	Shuttle vector series, multiple integrations; recycling of markers	Yeast	[166]
pMG	URA3, TRP1, LEU2, HIS3	6 kb	Single-crossover MultiSite Gateway compatible	Yeast	[167]
pRCC_K,	kanMX,	10 kb	CRISPR/Cas9 vector	Yeast	[148]
pRCC_N	natMX	10 kb	CRISPR/Cas9 vector	Yeast	[148]
pCRISPRyl_AXP	LEU2	12 kb	CRISPR/Cas9 vector	Yeast	[168]
pALS-1	*qa-2+*	13 kb	Based on the backbone of the mitochondrial plasmid of *N. crasssa* and the *E. coli* plasmid pBR325	Filamentous fungi	[150]
pALS-2	*qa-2+*	9 kb	Based on the backbone of the mitochondrial plasmid of *N. crasssa* and the *E. coli* plasmid pBR325	Filamentous fungi	[150]
pDV1001	*qa-2+*	11 kb	A hybrid pBR322 plasmid	Filamentous fungi	[151]
pAN7-1	*hph*	6 kb	High copy number	Filamentous fungi	[152]
pAN8-1	*ble*	6 kb	High copy number	Filamentous fungi	[153]
pPgpd-DsRed	*hph*	6 kb	Expression vector with a reporter protein	Filamentous fungi	[155]
pAg1-H3	*hph*	4 kb	Gene targeting and disruption, ATMT	Filamentous fungi	[157]
pWEF	*hph*	12 kb	pWEF32 undergoes homologous recombination, pWEF31 undergoes random recombination, ATMT	Filamentous fungi	[169]
pDESTR	*hph*	5 kb	Gene targeting and disruption, Gateway vector	Filamentous fungi	[170]
pCBGW-GFP	*hph*	8 kb	Gateway expression vector with GFP reporter gene	Filamentous fungi	[171]
pEX1	*pyrG*	10 kb	Vector with GFP reporter gene used in ATMT	Filamentous fungi	[172]
pEX2	*pyrG*	10 kb	Vector with DsRed reporter gene, ATMT	Filamentous fungi	[172]
pBI-hph	*hph*	15 kb	ATMT plasmid	Filamentous fungi	[173]
pLUO	*hph*	6 kb	Vector with a red (mCherry) or a green (eGFP) reporter protein ATMT plasmid	Yeast and Filamentous fungi	[174]
pDL11	*argB*	1 kb	Integration vector, synthetic biology	Filamentous fungi	[175]
pDL12	*argB*	1 kb	Integration vector, synthetic biology	Filamentous fungi	[175]
pDL13	*trpC*	1 kb	Integration vector, synthetic biology	Filamentous fungi	[175]
pDL14	*trpC*	1 kb	Integration vector, synthetic biology	Filamentous fungi	[175]
pDL15	*niaD*	1 kb	Integration vector, synthetic biology	Filamentous fungi	[175]
pBHt1	*hph*	8 kb	pCAMBIA vector series, used in ATMT	Filamentous fungi	[125]
pFungiway1	*hph*	5 kb	pCAMBIA vector series, Gateway expression plasmid used in ATMT	Filamentous fungi	[176]
pFungiway3	*G-418*	5 kb	pCAMBIA vector series, Gateway expression plasmid used in ATMT	Filamentous fungi	[176]
pFungiway5	*G-418*	8 kb	pCAMBIA vector series, Gateway repression plasmid used in ATMT	Filamentous fungi	[176]
pFungiway7	*hph, G-418*	8 kb	pCAMBIA vector series, Gateway repression plasmid used in ATMT	Filamentous fungi	[176]
pBIG2RHPH2	*hph*	9 kb	Constructed on the backbone of pBIN19 used in the ATMT	Filamentous fungi	[177]
pUR5750	*hph*	14 kb	Used in ATMT	Filamentous fungi	[121]
pAg1-hph	*hph*	3 kb	Binary vector used in ATMT	Filamentous fungi	[178]
pOSCAR	*hph*	9 kb	A vector developed with the method for One Step Construction of Agrobacterium-Recombination-ready-plasmids (OSCAR) with the Gateway technology and used in ATMT	Filamentous fungi	[136]
pFC330	*pyrG*	15 kb	AMA1 plasmid with *Aspergillus* optimized *cas9*. CRISPR/Cas vector	Filamentous fungi	[159]
pFC331	*argB*	15 kb	AMA1 plasmid with *Aspergillus* optimized *cas9*. CRISPR/Cas vector	Filamentous fungi	[159]
pFC332	*hph*	15 kb	AMA1 plasmid with *Aspergillus* optimized *cas9*. CRISPR/Cas vector	Filamentous fungi	[159]
pFC333	*ble*	15 kb	AMA1 plasmid with *Aspergillus* optimized Cas9. CRISPR/Cas vector	Filamentous fungi	[159]
pFC334	*argB*	15 kb	AMA1 plasmid with *Aspergillus* optimized Cas9, sgRNA expressed with ribozymes. CRISPR/Cas Vector	Filamentous fungi	[159]
Anep8_Cas9	*pyrG*	15 kb	AMA1 plasmid. *Aspergillus*-optimized Cas9 with LIC tags for easy gRNA cloning. CRISPR/Cas vector	Filamentous fungi	[160]
pAC1430	*pyrG*	15 kb	AMA1 plasmid with *Aspergillus* optimized Cpf1. CRISPR Vector	Filamentous fungi	[179]
pAC1748	*argB*	16 kb	AMA1 plasmid with *Aspergillus* optimized Cpf1. CRISPR Vector	Filamentous fungi	[179]
pAC1749	*hph*	15 kb	AMA1 plasmid with *Aspergillus* optimized Cpf1. CRISPR Vector	Filamentous fungi	[179]
pAC1750	*ble*	15 kb	AMA1 plasmid with *Aspergillus* optimized Cpf1. CRISPR Vector	Filamentous fungi	[179]
pFTK036	*amdS*	4.1 kb	Part Plasmid Entry Vector (LVL0) from the Fungal Modular Cloning ToolKit.	Filamentous fungi	[180]
pFTK038	*hph*	3.2 kb	Part Plasmid Entry Vector (LVL0) from the Fungal Modular Cloning ToolKit.	Filamentous fungi	[180]
pFTK061	n/a	6.3 kb	Part Plasmid Entry Vector (LVL0) from the Fungal Modular Cloning ToolKit. SpCas9 (for fusion).	Filamentous fungi	[180]
pFTK093	n/a	5.8 kb	Vector part (LVL1) from the Fungal Modular Cloning ToolKit. sgRNA transcription unit (MoClo lvl1 unit), P-gpdA-HH-sgRNA-HDV-Ttrpc, replacable LacZ gene	Filamentous fungi	[180]

* LEU2: 3-isopropylmalate dehydrogenase, URA3: orotidine-5′-phosphate decarboxylase, TRP1: phosphoribosylanthranilate isomerase, HIS3: Imidazoleglycerol-phosphate dehydratase, ADE2: phosphoribosylaminoimidazole carboxylase, HIS2: Histidinolphosphatase, ADE1: N-succinyl-5-aminoimidazole-4-carboxamide ribotide synthetase, MET15: O-acetyl homoserine-O-acetyl serine sulfhydrylase, HIS3: imidazoleglycerol-phosphate dehydratase, CAN1: arginine transporter Can1, LYS5: Phosphopantetheinyl transferase, *amds*: acetamidase gene, *ble*: bleomycin resistance gene, *dsd*: D-serine deaminase, *hph*: hygromycin resistance gene, *kan*: kanamycin resistance, *nat*: nourseothricin N-acetyl transferase, Kl.URA3: *Kluyveromyces lactis* URA3 selection marker, LYS2: -aminoadipate reductase, kanMX: geneticin resistance cassette, natMX: nourseothricin resistance cassette, *qa-2+:* 3-dehydroquinate hydrolyase, *pyrG*: Orotidine-5′-decarboxylase gene, *argB*: ornithine transcarbamylase, *trpC*: tryptophan C gene, *niaD*: nitrate reductase encoding gene, G-418: Geneticin resistance.

## Data Availability

Not applicable.
